# Evaluation of Single-Bundle versus Double-Bundle PCL Reconstructions with More Than 10-Year Follow-Up

**DOI:** 10.1155/2015/751465

**Published:** 2015-02-01

**Authors:** Masataka Deie, Nobuo Adachi, Atsuo Nakamae, Kobun Takazawa, Mitsuo Ochi

**Affiliations:** ^1^Department of Physical Therapy and Occupational Therapy Sciences, Graduate School of Health Sciences, Hiroshima University, 1-2-3 Kasumi, Minami-ku, Hiroshima 734-8553, Japan; ^2^Department of Orthopaedic Surgery, Graduate School of Biomedical Sciences, Hiroshima University, 1-2-3 Kasumi, Minami-ku, Hiroshima 734-8551, Japan

## Abstract

*Background*. Posterior cruciate ligament (PCL) injuries are not rare in acute knee injuries, and several recent anatomical studies of the PCL and reconstructive surgical techniques have generated improved patient results. Now, we have evaluated PCL reconstructions performed by either the single-bundle or double-bundle technique in a patient group followed up retrospectively for more than 10 years. *Methods*. PCL reconstructions were conducted using the single-bundle (27 cases) or double-bundle (13 cases) method from 1999 to 2002. The mean age at surgery was 34 years in the single-bundle group and 32 years in the double-bundle group. The mean follow-up period was 12.5 years. Patients were evaluated by Lysholm scoring, the gravity sag view, and knee arthrometry. *Results*. The Lysholm score after surgery was 89.1 ± 5.6 points for the single-bundle group and 91.9 ± 4.5 points for the double-bundle group. There was no significant difference between the methods in the side-to-side differences by gravity sag view or knee arthrometer evaluation, although several cases in both groups showed a side-to-side difference exceeding 5 mm by the latter evaluation method. *Conclusions*. We found no significant difference between single- and double-bundle PCL reconstructions during more than 10 years of follow-up.

## 1. Introduction

Posterior cruciate ligament (PCL) injuries are not rare in acute knee injuries; however, the treatment choice between conservative and surgical for PCL injury remains controversial. Recent advances in knowledge about PCL anatomy and biomechanical function [1–5] have increased research into surgical procedures of PCL reconstruction such as single-bundle [6–9], remnant-preserved [10], double-bundle [11], and tibial in-lay [12] techniques, with improved clinical results for patients; however, few studies have directly compared results between single- and double-bundle PCL reconstructions. We therefore undertook a clinical study of these two surgical techniques conducted at our institution with retrospective follow-up of more than 10 years. We hypothesized that double-bundle PCL reconstruction yielded better overall results than the single-bundle procedure.

Our principal aim was to evaluate the long-term results of PCL reconstruction following arthroscopic four-strand, single-bundle or 2 × 2-strand, double-bundle PCL reconstruction using hamstring tendons, based on clinical assessment. We report both objective and subjective outcomes and concentrate on high-grade PCL injuries that were refractory to an initial period of functional treatment.

## 2. Materials and Methods

The ethics committee of the Hiroshima University approved all protocols for this study, which was a retrospective observational analysis of a series of patients who underwent primary PCL reconstruction for grade III and associated surgeries. This was a single center study and all operations were performed or supervised by two surgeons using the same postoperative rehabilitation.

The indications for surgical reconstruction of PCL were magnetic resonance imaging (MRI) evidence of PCL rupture, symptoms of instability with normal daily activities, the inability to play sports, and symptoms being refractory to a period of nonoperative treatment. We performed 40 PCL reconstructions by either the single- or double-bundle method from 1999 to 2002. There were 27 single-bundle cases (18 males, 9 females) and 13 double-bundle cases (11 males, 2 females). The mean age at surgery was 34 years (range: 21–54 years) in the single-bundle group and 32 years (range: 23–52 years) in the double-bundle group. By gravity sag view, the side-to-side differences before surgery were 7–14 mm in single-bundle cases and 8–14.5 mm for the double-bundle reconstructions.

We also performed combined ligament reconstructions during the PCL reconstructions, involving 1 ACL reconstruction, 4 MCL reconstructions, and 4 PLC reconstructions in the single-bundle cases, and 1 ACL reconstruction, 2 MCL reconstructions, and 3 PLC reconstructions in the double-bundle cases. Finally, we examined 18 single-bundle cases involving 2 MCL reconstructions and 2 PLC reconstructions and 10 double bundle cases involving 1 MCL reconstruction and 2 PLC reconstructions. The follow-up ratio was 66.6% and 76.9% for single-bundle and double-bundle procedures, respectively. All cases were followed up for 2 years after surgery; however, at the final follow-up, 8 patients were not contactable and the other 4 patients had relocated such that they no longer attended our hospital for clinical evaluations. The mean follow-up period of the examined patients was 12.5 years (10–14 years). All patients were evaluated using the Lysholm score, the gravity sag view measured at 90 degrees of knee flexion to assess side-to-side differences, and knee arthrometry (KneeLax III, MR Systems, Haarlem, Netherlands) at 70 degrees of knee flexion.

### 2.1. Surgical Procedure

**Figure 1:**
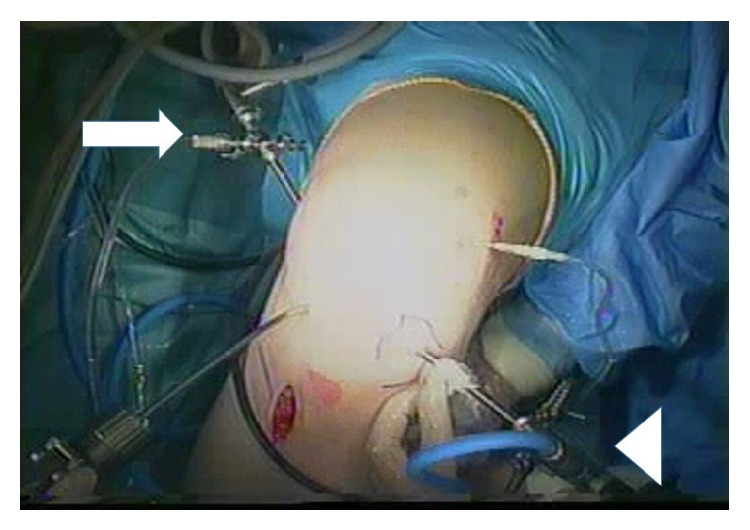
A two-camera system for PCL reconstruction. One camera (arrowhead) is used for the anterior approach and the other (arrow) is used for the posteromedial approach.

For the PCL reconstructions, we used double-vision arthroscopy (Figure 1). The surgical technique consisted of routine examination under anesthesia and arthroscopy using standard anterolateral and anteromedial portals to confirm the diagnosis and any other concomitant injuries. We create a posteromedial portal, using the original posteromedial portal guide system [13], to remove the torn PCL and to define the tibial PCL footprints. We then obtained more than 24 cm of hamstring tendon (gracilis tendon and semitendinosus tendon) through a vertical incision on the proximal medial tibia, to use as free grafts. Using a PCL drilling guide (Smith & Nephew, Andover, MA, USA), the tibial tunnel was made over an aiming guide pin, which was positioned on the tibial PCL footprint during the posterior tibia viewing using 30° arthroscopy through the posteromedial portal, with the drill sleeve positioned on the anteromedial aspect of the proximal tibia. Then the tibial bone tunnel was created at PCL footprint. At both single and double bundle techniques only one bone tunnel were made at the tibia. The tibial bone tunnels were drilled 0.5 mm over than the graft diameter of the tibial site. The graft diameters were measured using the sizing tubes every 0.5 mm. The tunnel edges were chamfered with rasps and a shaver.

For the single-bundle procedure, the femur tunnel was made in the intercondylar space 5 mm posterior to the articular margin and 5 mm distal from the Blumensaat line, which is closed to the anterolateral bundle insertion area. For the double-bundle procedure, the femur tunnel was made at the original attachments of anterolateral and posteromedial bundles.

**Figure 2:**
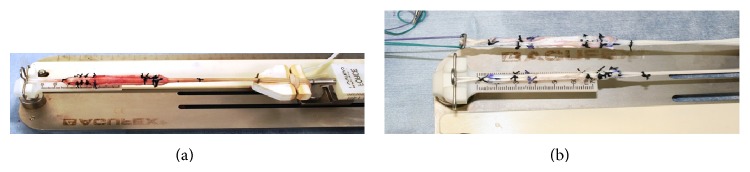
Our PCL substitutes made from the semitendinosus and gracilis tendons. (a) Substitute for single-bundle PCL reconstruction. (b) Substitute for double-bundle PCL reconstruction.

We used hamstring tendons as grafts to reconstruct the PCL in all cases. A minimum graft length of 8 cm is necessary to ensure a complete reconstruction. For the single-bundle method, we made four bundle substitutions using semitendinosus and gracilis tendons in the single bundle on the femur side, resulting in a graft diameter >8.5 mm (Figure 2(a)). For the double-bundle method, we made two × two bundle substitutes using the hamstring tendons, with one of the graft diameters >6 mm (Figure 2(b)). The substitutes were fixed with double staples at the front of the tibia.

### 2.2. Postoperative Rehabilitation

The postoperative rehabilitation program was the same for both surgical procedures. Until 2 weeks after the surgery, the knee was fixed with a knee brace, after which a PCL brace was applied. At 6 months after surgery, the PCL brace was removed and the patients were allowed to commence jogging at 6 months, with sporting activities introduced at 10–12 months.

### 2.3. Statistical Analysis

These study data were analyzed by two-way ANOVA, with a* P* value less than 0.05 considered significant.

## 3. Results

### 3.1. Lysholm Scores

The mean score for single-bundle reconstructions was 89 points at 2 years after surgery and 82 points at 10 years after surgery, and for the double-bundle reconstructions it was 92 points at 2 years after surgery and 83 points at 10 years after surgery. Both scores were slightly decreased due to knee pain.

**Figure 3:**
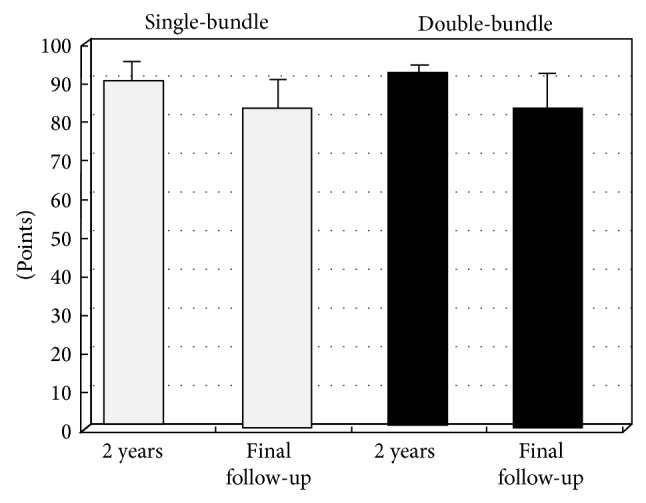
This graph shows the Lysholm scores. There were no significant differences between single- and double-bundle procedures or between results at 2 years and >10 years. The error bars show SD.

There were no significant differences between the two reconstruction methods at either 2 or 10 years after surgery (Figure 3). Of the 18 single-bundle cases and 10 double-bundle cases, 7 and 6 patients, respectively, felt occasional knee pain. On X-ray, all cases across both groups showed advanced osteoarthritis changes compared to the opposite knee.

### 3.2. Gravity Sag View

**Figure 4:**
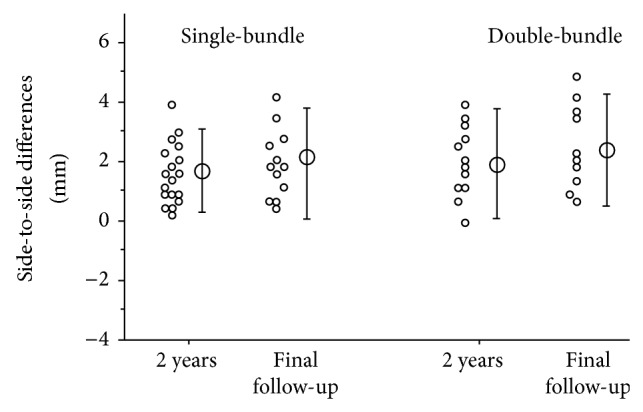
This graph shows the side-to-side* differences* by gravity sag view. The data represent operated side* values* minus the opposite side. There were no significant differences between single- and double-bundle procedures or between results at 2 years and >10 years.

The gravity sag view revealed side-to-side differences of 1.8 mm (mean value) in the single-bundle group at 2 years, while after 10 years it had increased to 2.5 mm. The mean differences in the double-bundle group were 2.0 mm at 2 years and 2.6 mm at 10 years after surgery—this also represented an increase with longer follow-up (Figure 4), although the differences between surgical procedures were not significant.

### 3.3. Knee Arthrometry

**Figure 5:**
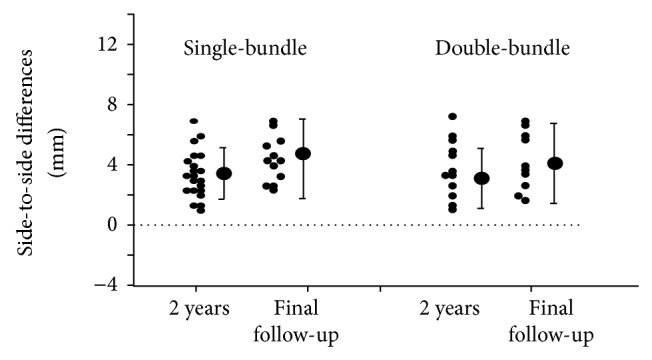
This graph shows the side-to-side differences by knee arthrometry at 133 N (30 pounds) in the knee at 70° flexion. The data represent operated side values minus the opposite side. There were no significant differences between single- and double-bundle procedures or between results at 2 years and >10 years.

The knee arthrometer testing found mean side-to-side differences in the single-bundle and double-bundle groups of 3.8 mm and 3.6 mm at 2 years and 4.5 mm and 4.3 mm at 10 years, respectively, after surgery. Both groups thus showed an increased difference with term of follow-up (Figure 5) and included several cases showing a side-to-side difference exceeding 5 mm (4 single-bundle cases (22.2%) and 3 double-bundle cases (30%)). None of these differences were significant.

## 4. Discussion

Treatment of PCL injuries remains a challenging clinical problem. Recently, double-bundle PCL reconstructions were reported to anatomically and functionally mimic the normal PCL, whereby anterolateral and posterolateral bundles may act separately to provide partial functional responsibility for joint stability [5].

In this paper we showed no clinically different results of significance between single- and double-bundle PCL reconstructions procedures, even at more than 10 years after surgery. This result did not support our original hypothesis that the double-bundle PCL reconstruction conferred more advantages than the single-bundle technique, an opinion also held by other orthopedic surgeons. While almost all cases had good clinical results overall, we also showed some cases of remaining instability with both surgical procedures and that neither could prevent advanced osteoarthritis visible on X-ray.

Our evaluation of the two procedures by Lysholm score, as a clinical and objective measure, and of the posterior instability by sag view and knee arthrometry demonstrated no significant differences between the groups and confirmed some cases with remaining instability. The results also confirm that PCL procedures by the two techniques studied here compare favorably with ACL reconstruction procedures, which achieve approximately 90% satisfactory clinical results [14].

The study results did highlight two problems with these types of PCL reconstructions. One is the femoral fixation site. The cortex of the proximal medial femoral condyle, at which the Endobutton is usually fixed, is anatomically thin and mechanically weak when the direction of the femoral tunnel is created towards the distal end of the femur to avoid the killer turn. The second is the killer turn problem. In the PCL reconstruction using the bone tunnel technique, the killer turns of the graft at the openings of the tibial and femoral bone tunnels are highlighted. Because the killer turns of the graft increase the mechanical stress to the exit of the bone tunnels or graft, they could cause tunnel enlargement or graft failure. One solution for this tibial problem is using the inlay technique to fix the tibial site, if the bone-patella tendon-bone is used for the graft [15]. These papers recommended early or aggressive rehabilitation after ligament reconstruction surgeries. However, another solution is slow rehabilitation, which we recommend based on the current study, such that PCL reconstruction patients avoid early obtainment of the flexion angle inducing posterior instability of the knee joint. Overall, we showed good clinical results in over 70% of patients and thus speculate that both of the clinical problems noted here arose during the rehabilitation programs. We propose that the flexion angle at 6 months after reconstruction should be around 110° in the case of relatively young patients as long as the substitute is a hamstring tendon rather than the bone-patella tendon-bone.

There were several limitations in this study. One is the retrospective nature of the evaluations. When we performed the relevant procedures, there were no clear indications for selecting between single- and double-bundle PCL reconstructions. Therefore, we examined cases for which the surgical procedures would bring the better clinical results. The second is the relatively low number of cases enrolled and the low follow-up rate. Finally, these cases included some combined ligament injuries, and it would be preferable to study prospectively isolated PCL injuries to determine the optimal treatment.

In conclusion, the PCL reconstructions evaluated herein achieved relatively good clinical results based on posterior stability, range of motion, and Lysholm scoring. However, we must solve several problems with these surgical procedures and the follow-up to obtain more satisfactory clinical and long-term results in the future.

## References

[1] Gollehon D. L., Torzilli P. A., Warren R. F. (1987). The role of the posterolateral and cruciate ligaments in the stability of the human knee. A biomechanical study. *The Journal of Bone and Joint Surgery—American Volume*.

[2] Grood E. S., Hefzy M. S., Lindenfield T. N. (1989). Factors affecting the region of most isometric femoral attachments. Part I. The posterior cruciate ligament. *The American Journal of Sports Medicine*.

[3] Ochi M., Murao T., Sumen Y., Kobayashi K., Adachi N. (1999). Isolated posterior cruciate ligament insufficiency induces morphological changes of anterior cruciate ligament collagen fibrils. *Arthroscopy*.

[4] Murao T., Ochi M., Jitsuiki J., Ikuta Y. (1997). The adverse effects of sectioning the posterior cruciate ligament in rabbits: changes in the structural and morphological properties of the femur-anterior cruciate ligament-tibia complex. *Archives of Orthopaedic and Trauma Surgery*.

[5] Amis A. A., Gupte C. M., Bull A. M. J., Edwards A. (2006). Anatomy of the posterior cruciate ligament and the meniscofemoral ligaments. *Knee Surgery, Sports Traumatology, Arthroscopy*.

[6] Kim S.-J., Kim H.-K., Kim H.-J. (1999). Arthroscopic posterior cruciate ligament reconstruction using a one-incision technique. *Clinical Orthopaedics and Related Research*.

[7] Jackson W. F. M., van der Tempel W. M., Salmon L. J., Williams H. A., Pinczewski L. A. (2008). Endoscopically-assisted single-bundle posterior cruciate ligament reconstruction: results at minimum ten-year follow-up. *The Journal of Bone and Joint Surgery—British Volume*.

[8] Adachi N., Ochi M., Uchio Y., Iwasa J., Ishikawa M., Shinomiya R. (2007). Temporal change of joint position sense after posterior cruciate ligament reconstruction using multi-stranded hamstring tendons. *Knee Surgery, Sports Traumatology, Arthroscopy*.

[9] Kim Y.-M., Lee C. A., Matava M. J. (2011). Clinical results of arthroscopic single-bundle transtibial posterior cruciate ligament reconstruction: a systematic review. *The American Journal of Sports Medicine*.

[10] Kim S. J., Kim S. H., Chun Y. M., Hwang B. Y., Choi D. H., Yoon J. Y. (2012). Clinical comparison of conventional and remnant-preserving transtibial single-bundle posterior cruciate ligament reconstruction combined with posterolateral corner reconstruction. *The American Journal of Sports Medicine*.

[11] Yoon K. H., Bae D. K., Song S. J., Lim C. T. (2005). Arthroscopic double-bundle augmentation of posterior cruciate ligament using split Achilles allograft. *Arthroscopy*.

[12] Kim S.-J., Choi C.-H., Kim H.-S. (2004). Arthroscopic posterior cruciate ligament tibial inlay reconstruction. *Arthroscopy*.

[13] Ochi M., Adachi N., Sumen Y., Uchio Y., Iwasa J. (1998). A new guide system for posteromedial portal in arthroscopic knee surgery. *Archives of Orthopaedic and Trauma Surgery*.

[14] Shelbourne K. D., Gray T. (2009). Minimum 10-year results after anterior cruciate ligament reconstruction. *The American Journal of Sports Medicine*.

[15] Seon J. K., Song E. K. (2006). Reconstruction of isolated posterior cruciate ligament injuries: a clinical comparison of the transtibial andtibialinlay techniques. *Arthroscopy*.

